# Autism spectrum disorder diagnosis using a new panel of immune- and inflammatory-related serum biomarkers: A case-control multicenter study

**DOI:** 10.3389/fped.2023.967954

**Published:** 2023-02-21

**Authors:** Benjamin Gesundheit, Philip David Zisman, Leah Hochbaum, Yehudit Posen, Avraham Steinberg, Gerald Friedman, Hersh D. Ravkin, Eitan Rubin, Ouriel Faktor, Ronald Ellis

**Affiliations:** ^1^ Cell-El Ltd., Jerusalem, Israel; ^2^ Shaare Zedek Medical Center, Jerusalem, Israel; ^3^ Department of Pediatrics Mackenzie Health, Children's Treatment Network, Diagnostic Autism Clinical Services, Ontario, Canada; ^4^ Shraga Segal Department of Microbiology, Immunology and Genetics, Ben-Gurion University of the Negev, Be'er Sheva, Israel; ^5^ Faktor Life Sciences & Diagnostics Consultations, Rehovot, Israel

**Keywords:** autism spectrum disorder, immunological biomarkers, diagnostics, monitoring, autoimmune

## Abstract

**Background and objectives:**

Children with autism spectrum disorder (ASD) present with distinctive clinical features. No objective laboratory assay has been developed to establish a diagnosis of ASD. Considering the known immunological associations with ASD, immunological biomarkers might enable ASD diagnosis and intervention at an early age when the immature brain has the highest degree of plasticity. This work aimed to identify diagnostic biomarkers discriminating between children with ASD and typically developing (TD) children.

**Methods:**

A multicenter, diagnostic case-control study trial was conducted in Israel and Canada between 2014 and 2021. In this trial, a single blood sample was collected from 102 children with ASD as defined in Diagnostic Statistical Manual of Mental Disorders [DSM)-IV (299.00) or DSM-V (299.00)], and from 97 typically developing control children aged 3–12 years. Samples were analyzed using a high-throughput, multiplexed ELISA array which quantifies 1,000 human immune/inflammatory-related proteins. Multiple logistic regression analysis was used to obtain a predictor from these results using 10-fold cross validation.

**Results:**

Twelve biomarkers were identified that provided an overall accuracy of 0.82 ± 0.09 (sensitivity: 0.87 ± 0.08; specificity: 0.77 ± 0.14) in diagnosing ASD with a threshold of 0.5. The resulting model had an area under the curve of 0.86 ± 0.06 (95% CI: 0.811–0.889). Of the 102 ASD children included in the study, 13% were negative for this signature. Most of the markers included in all models have been reported to be associated with ASD and/or autoimmune diseases.

**Conclusion:**

The identified biomarkers may serve as the basis of an objective assay for early and accurate diagnosis of ASD. In addition, the markers may shed light on ASD etiology and pathogenesis. It should be noted that this was only a pilot, case-control diagnostic study, with a high risk of bias. The findings should be validated in larger prospective cohorts of consecutive children suspected of ASD.

## 1. Introduction

Autism spectrum disorder (ASD) is a heterogeneous group of neurodevelopmental disorders presenting in early childhood, with an estimated prevalence of 0.7%–2.6% (1). The disorder affects social interaction and communication skills, and may present with unusual repetitive behaviors. Individuals with ASD often have co-morbidities, including epilepsy, depression, anxiety and attention deficit hyperactivity disorder. ASD typically persists for life and bears major social and financial implications on patients, family, society and healthcare systems (2). Available pharmacological and interventional therapies have shown only limited efficacy.

Behavioral features of ASD define its clinical phenotype. Diagnosis is made using the Diagnostic Statistical Manual (DSM-5) and is based on various tests, including the Autism Diagnostic Observation Schedule (ADOS) which has emerged as the reference standard in many ASD clinics. Many of these tests are less sensitive in very young children, thus precluding early diagnosis. Moreover, these tests are very time-consuming and require multiple visits and many hours with a professional to administer them and analyze the results. Finally, the subjective nature of these tests limits their utility due to frequent false-positive and false-negative diagnoses. Thus, an objective test for ASD based on quantitative biomarkers would be very valuable for improving the accuracy and efficiency of diagnosis.

Despite intensive searches for objective biomarkers (3), no objective diagnostic biomarker-based test has been established for clinical use. Quantification of biological markers significantly associated with ASD could be useful for diagnostic accuracy and might correlate with clinical severity and prognosis. Such objective biomarkers may also facilitate the selection of patients who may benefit from specific treatments.

Autoimmune activities with chronic neuro-inflammation have been suggested as factors contributing to the etiology of some ASD cases (4). Several large studies have shown significant associations between ASD and family history of autoimmune disease (5), although the percent of ASD cases with a link to autoimmunity or inflammation is unknown. Since no biomarkers have been identified to support this link, the etiology and neuropathology of ASD remains elusive in general or at least in this subpopulation. In this study, we searched for immune/inflammation-related biomarkers in children with ASD in order to establish an objective ASD diagnostic assay.

## 2. Materials and methods

### 2.1. Group selection and recruitment of participants

This was a case-control multicenter study in ASD clinics, performed in 2014–21 in Israel and Canada. Following ethics approval (Shaarei Zedek Medical Center, Jerusalem, Israel, approval number 0164-19), venous blood samples were drawn from 102 children with ASD and from 97 typically developing (TD) control children at multiple sites. Treating physicians approached families of ASD children who met the following inclusion criteria: age 3–12 years and documented ASD diagnosed according to Diagnostic and Statistical Manual of Mental Disorders (DSM)-IV (299.00) or DSM-V (299.00). TD children were aged 3–12 years with no signs or family history of ASD. Exclusion criteria included mild infection (cold/fever/antibiotics) during the preceding month, severe convulsive disorder, serious infection or use of systemic steroids or cancer treatments during the preceding 6 months, or a sibling involved in the study. Signed informed consent was obtained from parents before any study procedures were initiated. A medical history questionnaire was filled out before blood collection.

### 2.2. Serum collection

Venous blood (5 ml) was collected into Vacutainer Blood Collection Serum Tubes (BD Becton Dickinson Ltd.), 500 µl of which were used to determine the complete blood count (CBC). The remaining blood was allowed to clot for 30 min at room temperature, then centrifuged for 10 min at 10,000 rpm, after which serum was aliquoted and stored immediately at −80°C. Subjects were not required to be fasting at the time of the blood draw.

### 2.3. Biomarker analysis

The serum was analyzed by Quantibody Human Cytokine Antibody Array X00 (# QAH-CAA-X00, RayBiotech Ltd., Atlanta, GA, USA), a multiplexed sandwich enzyme-linked immunosorbent assay (ELISA)-based quantitative array platform that includes a combination of 25 non-overlapping arrays of 40 immune-/inflammation-related protein markers each, to quantify 1,000 human protein markers. Each sample was tested in quadruplicate for each biomarker.

### 2.4. Sample size justification

A total of 194 participants (97 per group) were requested to estimate 80% of sensitivity or specificity with 95% level of confidence and an absolute error of 8%, in a case control study. Sample size was calculated using Mark Stevenson (2022). epi.ssdxsesp: A package for analyzing epidemiological data. R package version 2.0.54 URL: https://cran.r-project.org/package=epi.ssdxsesp.

### 2.5. Statistical analysis

#### 2.5.1. Training/testing sets

A standard 10-fold cross-validation (10xCV) procedure was used. Data were divided into 10 sets maintaining the ASD/TD ratios, with each case assigned to 1 of 10 folds (i.e., 19–20 subjects per fold, randomly chosen in a class-preserving procedure). Each fold was used as a hold-out set, training a multiple logistic regression (MLR) model on the remaining 90%. The resulting model was evaluated on the held-out fold, obtaining an estimated performance of the model. As this procedure was repeated for each fold, 10 models were created, each with its own performance; the result reflects the average (standard deviation) of the 10 models. Since training was based on 90% of the data, the models were trained on partially overlapping data. On the other hand, the tests were conducted on unique data sets.

#### 2.5.2. Feature selection

To select features, any feature meeting one of two criteria was removed: (1) “Mostly zero” features (i.e., features with more than P1 zeroes, where P1 is parameter #1) were discarded from analysis; (2) Feature correlated to other features and not chosen as representative.

1. Any feature with a high frequency of 0 values was eliminated. Only training data were used for this step.
2. For correlation clustering (6), a graph was created connecting any two features with a Spearman correlation coefficient (*R*^2^) ≥P2, where P2 is parameter #2, using P2 = 0.5 unless otherwise specified). The graph was traversed to transitively define clusters (i.e., if feature A was connected feature B and feature B belonged to cluster X, then feature A was assigned to cluster X). For all features assigned to the same correlation cluster, the feature with the highest mean correlation with all other members of the cluster was chosen as the cluster representative. All other features in the cluster were eliminated. Only training data were used for this step.

These steps were applied sequentially, i.e., the second filter was only run on features that passed the first filter. The remaining features were ranked by their ability to perform as a single marker for predicting if a patient is ASD or TD using the sklearn.feature_selection.f_regression function on the test set of each fold. After ranking, the top 1% of the features (10 features) were selected. The features selected for each fold were always included in the MLR model; the algorithm only seeks those coefficients giving the best separation between ASD and TD cases. We arbitrarily chose to consider only 1% of the features, since logistic regression models with too many features are generally difficult to use and interpret in a clinical setting.

#### 2.5.3. Building multiple logistic regression models

MLR is based on multiple logistic regression, optimizing the coefficients of a logistic formula to optimize its fit to the observed subjects’ status. In other words, the method finds the best logistic formula that predicts the status of patients from their features. We used the method described above (under feature selection) to choose the markers to be considered. All chosen features were used in the MLR model (an “all in” approach). Note that all MLR models were developed on normalized (Z-transformed) data, since bringing all features to the same range facilitates interpretation of the coefficients.

#### 2.5.4. Statistical tests and critical values

To compare marker values between ASD cases and controls the student's *t*-test with the Benjamini-Hochberg algorithm to control for false discovery in multiple testing (7) was used. Unless specified otherwise, a *p*-value cutoff of 0.05 was used. For independence tests, the chi-square test was used.

Accuracy, sensitivity and specificity analyses were conducted using 0.5 as a critical value (i.e., MLR values of 0.5 and above were taken to indicate ASD and value under 0.5 were used to indicate TD). For logistic regression, a cutoff of 0.5 was arbitrarily chosen as is customary.

## 3. Results

**Figure 1:**
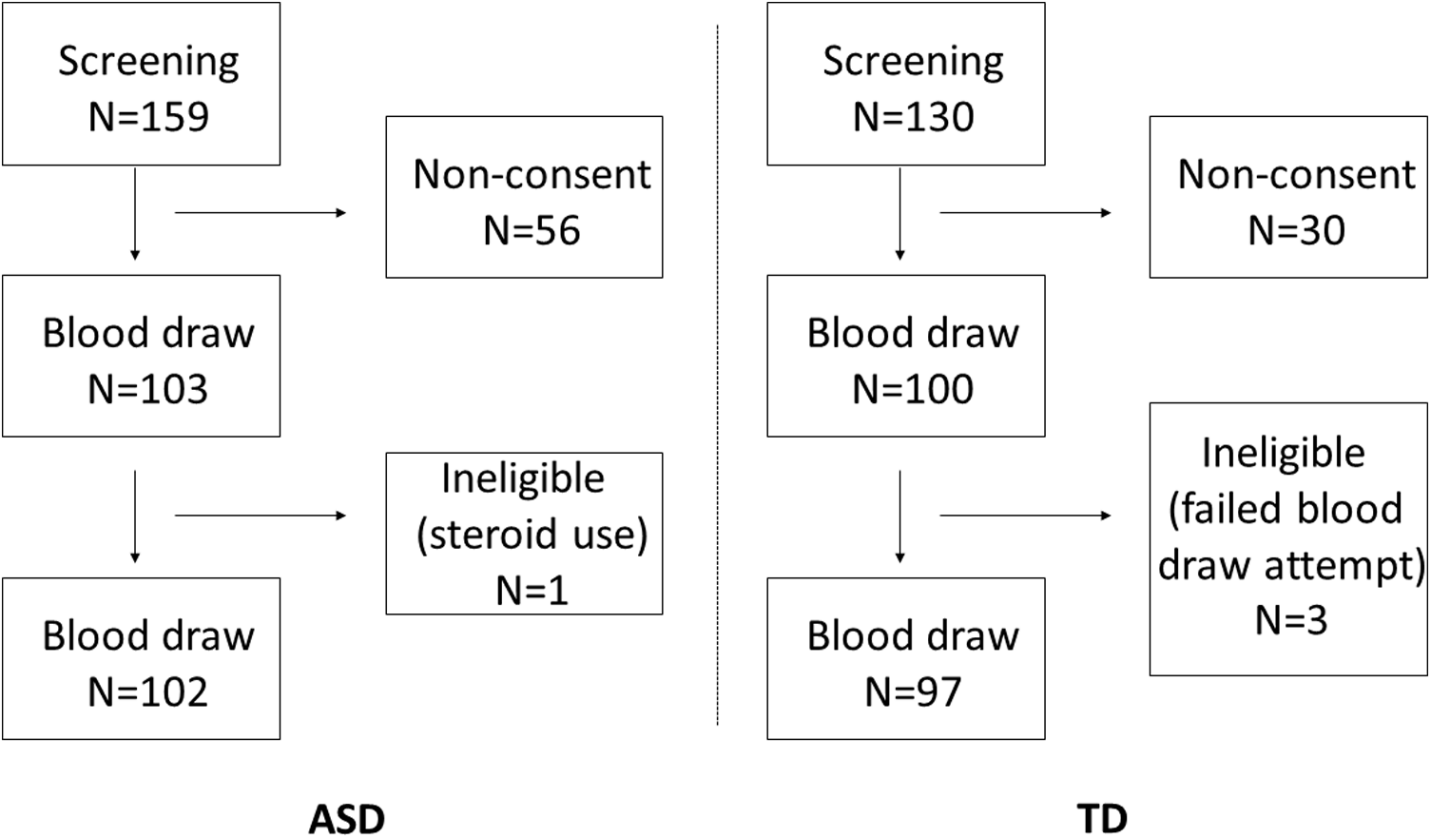
Flowchart of disposition of subjects in each study arm.

Parents of 159 ASD children were contacted for potential recruitment; 103 provided their consent and blood samples were collected. After blood draw, one child was found ineligible due to steroid use. A total of 130 parents of TD children were contacted; 100 provided their consent. In 3 children, blood sampling failed, leaving the TD cohort with a total of 97 samples. A subject disposition flow chart is provided in Figure 1. A summary of participant demographics and medical history is presented in Table 1. Most participating children were male, and most were native-born Israelis. A greater percentage of ASD vs. TD children had a sibling (16.5% vs. 0%) or relative (29.7% vs. 6.2%) with ASD. Food allergies were reported for 16.5% of the ASD as compared to 3.1% of the TD children and more of the ASD children had a mother and/or father with an autoimmune condition (13.2% and 9.9%, respectively) as compared to TD children (1.0% and 1.0%, respectively). A larger proportion of ASD children were delivered in a Caesarean section (31.2% vs. 6.2%), or after a complicated pregnancy or labor (33.0% vs. 7.2%). Use of prescription medications during pregnancy was reported by 34.0% of the mothers of ASD children as compared to 9.3% of the mothers of TD children.

**Table 1:** Demographics and medical history of study participants.

	ASD (*N* = 102)	TD (*N* = 97)
**Gender, *n* (%)**		
Male	89 (87.3)	60 (61.9)
Female	11 (10.8)	37 (37.8)
**Age (years), mean (SD)**		
Male	5.2 (2.0)	6.8 (2.7)
Female	5.6 (2.6)	7.1 (2.3)
**Country of birth, *n* (%)**		
Israel	82 (80.4)	86 (88.7)
Other	20 (19.6)	11 (11.3)
**Paternal country of birth, *n* (%)**		
Israel	51 (50)	61 (62.9)
Former USSR	14 (13.7)	5 (5.2)
North America	7 (6.8)	17 (17.6)
United Kingdom	2 (2)	5 (5.2)
Other	6 (5.9)	5 (5.2)
Unknown	22 (21.6)	4 (4.1)
**Maternal country of birth, *n* (%)**		
Israel	48 (47.1)	62 (63.9)
Former USSR	17 (16.7)	11 (11.3)
North America	10 (9.8)	18 (18.6)
United Kingdom	1 (1.0)	2 (2.1)
Other	9 (8.8)	2 (2.1)
Unknown	17 (16.7)	2 (2.1)
Food or medicine allergy^a^, *n* (%)	15 (16.5)	3 (3.1)
Delivered in C-section^a^, *n* (%)	29 (31.2)	6 (6.2)
Significant complications during pregnancy/labor/immediately after birth^a^, *n* (%)	30 (33.0)	7 (7.2)
Mother used prescription medicine during pregnancy^a^, *n* (%)	31 (34.0)	9 (9.3)
Mother with autoimmune condition^a^, *n* (%)	12 (13.2)	1 (1.0)
Father with autoimmune condition^a^, *n* (%)	9 (9.9)	1 (1.0)
Sibling with ASD^a^, *n* (%)	15 (16.5)	0 (0)
Relative with ASD^a^, *n* (%)	27 (29.7)	6 (6.2)

ASD, autism spectrum disorder.

^a^ For the ASD group, data were only available for 91/102 children.

**Figure 2:**
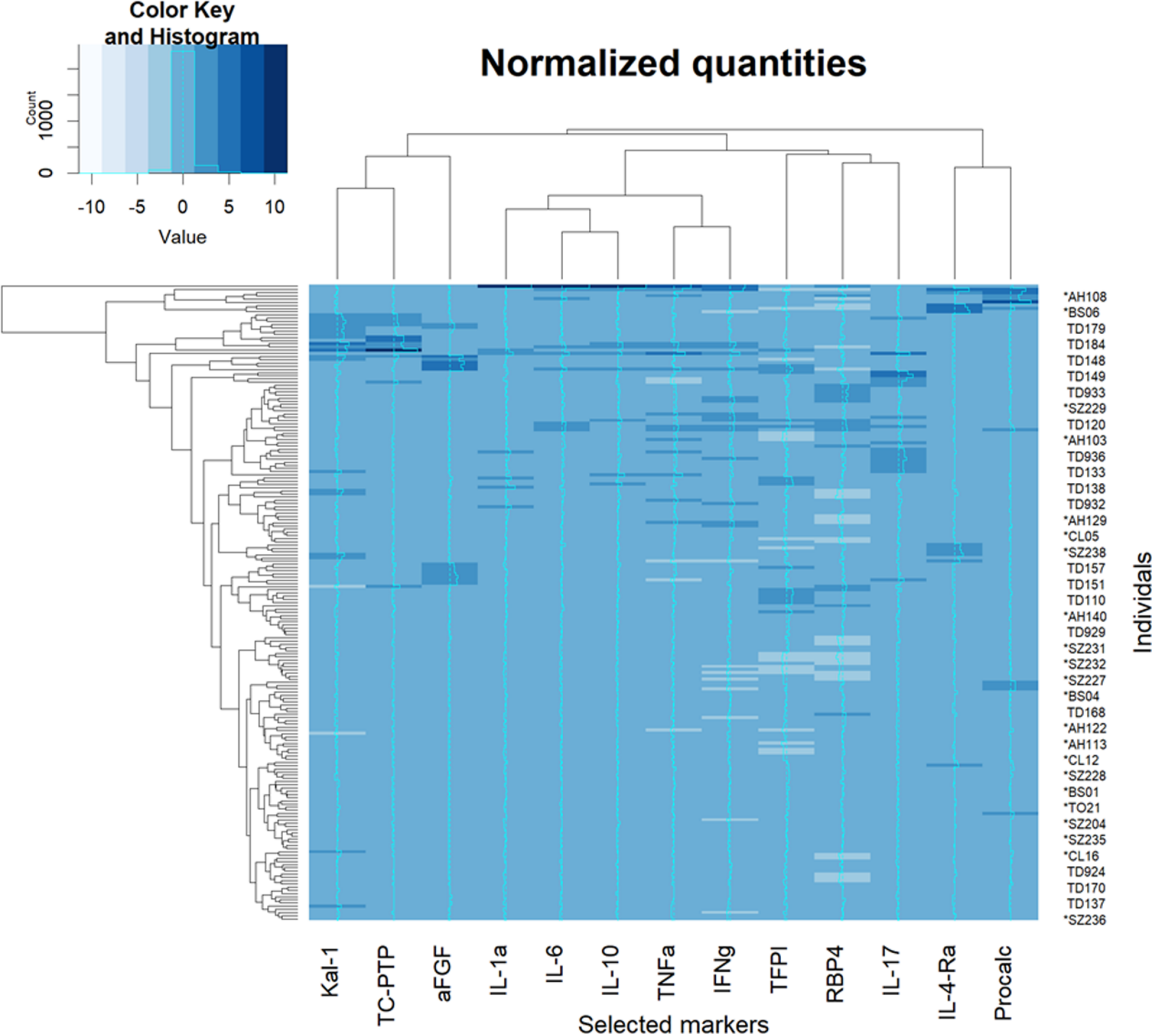
Heatmap of raw measurements. A heatmap of Z-normalized levels of the markers selected to be included in multiple logistic regression. An intensity curve is provided for each marker (a cyan in the middle of each column). Both rows and columns were clustered with hierarchical clustering and rearranged for clarity. Procalc, procalciton; Kal-1, kallikrein-1.

**Figure 3:**
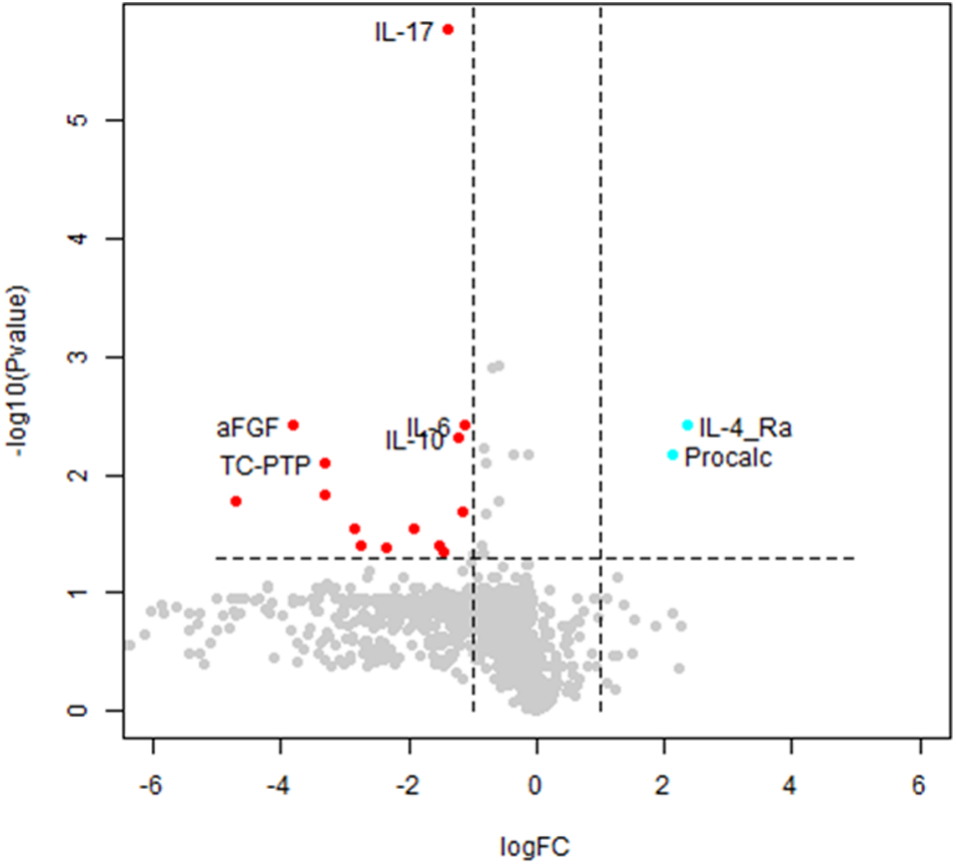
Volcano plot of autism spectrum disorder (ASD) cases vs. typically developing (TD) controls. A volcano plot of the *t*-test differences between ASD and TD children. Each dot represents a marker. Markers significantly downregulated in ASD with a change of 2-fold or more are shown in red. Significantly altered markers increased 2-fold or more in ASD are shown in cyan. Markers chosen to be included in any MLR model are labelled. FC, fold change; Procalc, procalcitonin.

**Figure 4:**
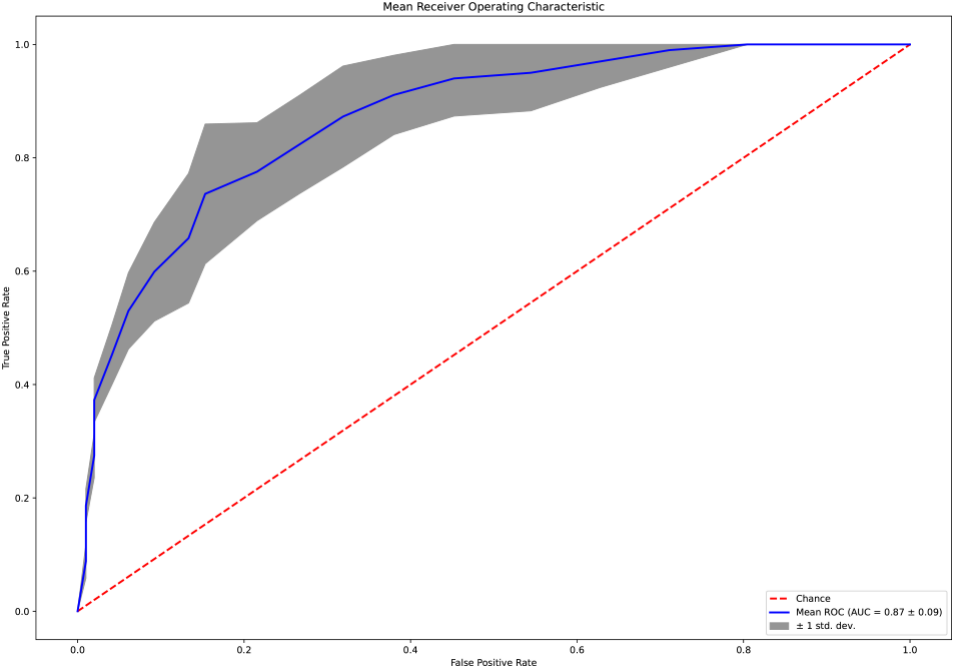
Receiver operator curve of the MLR model.

The normalized (Z-transformed) raw measurements of selected genes are shown in Figure 2, which shows some measurements to be different in some samples but not in others. Some of these differences were highly significant, as can be observed in the volcano plot of the difference between ASD and control (Figure 3). This plot shows a clear bias toward reduction in ASD children (Figure 3). Application of the two feature selection steps, i.e., the “mostly zeros” filter and the “correlation clustering” filter, eliminated 86 ± 3 and 107 ± 4 features, respectively, in each of the 10 folds (note that the correlation clustering filter was only applied to features that passed the mostly zeros filter). MLR analysis revealed 12 immunological markers that recurred more than once (Table 2). The levels of interleukin (IL)-17, acidic fibroblast growth factor (aFGF), interferon (IFN)-γ, and tumor necrosis factor (TNF)-α were included in all 10 models, and the levels of IL-4Ra, IL-6 and IL-1a were included in at least half of the MLR models, while procalcitonin, T cell protein tyrosine phosphatase (TC-PTP), tissue factor pathway inhibitor (TFPI), retinol binding protein 4 (RBP4) and kallikrein1 were included in four, three, three, two and two models, respectively. Since the feature selection process was performed separately for each step, 4 markers were chosen in all 10 folds, and 8 more markers were selected more than once. Most of the markers included in all 10 models, as well as many of the markers included in fewer models, have been reported to be associated with ASD (Table 2). Receiver-operator curve (ROC) analysis suggested that the results were much better than expected randomly: the area under the curve (AUC) of the MLR model was 0.86 ± 0.06, compared to 0.5 expected by chance (Figure 4). No significant difference in prediction accuracy was found by gender or sample source (*p* = 0.06 and 0.16, Chi-squared test).

**Table 2:** Identified diagnostic biomarkers and their potential biological role in autism spectrum disorder.

	Marker (# of MLR recurrence)	Biological role in ASD	*p*	FC
1.	IL-17 (10)	Levels of IL-17 are elevated in some ASD children (8). IL-17 is associated with various neurological and autoimmune disorders (9). Mice exposed to IL-17 during gestation have autism-like behaviors (10).	2E-06	−1.4
2	FGFa; FGF1 (10)	Fibroblast growth factor 1 (FGF1) regulates cell proliferation, cell division and neurogenesis, with possible association to autism (10).	0.004	−3.8
2.	IFN-g (10)	Levels of IFN-g are dysregulated in some autistic children (11).	0.001	−0.7
3.	IL-10 (10)	The ratio of a common proinflammatory cytokine to an anti-inflammatory cytokine (IL-10) has been found to be important in ASD (12).	0.005	−1.2
4.	TNF-a (10)	Levels are dysregulated in ASD (13, 14).	0.001	−0.6
5.	IL-4Ra (9)	Interleukin 4 receptor. High IL-4 levels are associated with ASD (15, 16).	0.004	2.4
6.	IL-6 (8)	Levels are dysregulated in ASD (13, 17, 18).	0.003	−1.1
7.	IL-1a (6)	Associated with Alzheimer’s disease and multiple sclerosis (19). No information on association with ASD.	0.006	−0.8
8.	Procalcitonin (4)	Inflammatory marker in schizophrenic patients (20). No information on association with ASD.	0.007	2.1
9.	TC PTP (3)	T-cell protein tyrosine is highly expressed in hematopoietic tissues. No information on association with ASD.	0.008	−3.3
10.	TFPI (3)	Tissue factor pathway inhibitor. No information on association with ASD.	0.007	−0.4
11.	RBP4 (2)	Decreased concentration is associated with the presentation of the autistic regression phenomenon (21).	0.007	−0.1
12.	Kallikrein 1 (2)	A subgroup of serine proteases capable of cleaving peptide bonds. No information on association with ASD.	0.008	−0.8

Shown are the recurring markers (occurring in 2 out of 10 or more folds), their multiple testing-adjusted *t*-test *p*-values comparing ASD to TD control children and the logged (base 2) fold change (FC) in mean values between ASD and TD control children.

The equations generated by MLR (see Supplementary File) consider joined-markers-based prediction and not individual prediction of a single marker. Since only those features most strongly associated with ASD were used as a single marker, most of the features included in the MLR equations were among the best single markers.

The performance characteristics of the model (Table 3) feature overall assay accuracy of 0.82 ± 0.09 (sensitivity 0.87 ± 0.08; specificity: 0.77 ± 0.14), which is significantly better than the accuracy expected by chance (i.e., 0.52), which can be obtained from always guessing the majority class (*p* = 10^−6^, one-sample *t*-test).

**Table 3:** Performance of the multiple logistic regression (MLR) model in 10 folds.

	F (P1)	F (P2)	Accuracy^a^	Sensitivity^a^	Specificity^a^	F1 score^a^
MLR1	89	105	0.80	0.82	0.78	0.82
MLR2	82	107	0.75	0.91	0.55	0.80
MLR3	85	101	0.70	0.80	0.60	0.73
MLR4	86	107	0.95	1.00	0.90	0.95
MLR5	87	109	0.85	0.90	0.80	0.86
MLR6	82	107	0.80	0.90	0.70	0.82
MLR7	88	109	0.90	0.90	0.90	0.90
MLR8	82	108	0.85	0.90	0.80	0.86
MLR9	86	114	0.70	0.70	0.70	0.70
MLR10	90	103	0.95	0.90	1.00	0.95
**Total**	**85.7 ± 3.0**	**107.0 ± 3.6**	**0.82 ± 0.09**	**0.87 ± 0.08**	**0.77 ± 0.14**	**0.84 ± 0.08**

F(P1): the number of features removed by “mostly zero” filtering (denoted as). F(P2): the number of features removed by the “correlation clustering” method of the features remaining after “mostly zero” filtering. Total: the summary statistics for 10-fold cross validation, providing the mean ± standard deviation for the 10 folds.

^a^ Using a cutoff of 0.5 to define ASD.

## 4. Discussion

Using a multiplexed sandwich ELISA-based array platform for simultaneous determination of the concentration of ∼1,000 immunological biomarkers, we identified 12 biomarkers that provide an overall ASD diagnostic accuracy of 82%. Of the 102 ASD children included in the study, 13% were negative for all these biomarkers; these cases may represent a subgroup of children with non-immune-associated ASD or with other biomarkers not used in the multiplex array. Further research to identify additional immune-associated biomarkers is ongoing. A recent study of blood samples showed immune-related biomarkers associated with molecules important for brain development (22).

The Quantibody Human Cytokine Antibody Array used in this work combines the advantages of high detection sensitivity and specificity of ELISA with high assay throughput. The assay enables simultaneous measurement of 1,000 proteins in serum, a most readily available tissue, and is array-based, thus rendering it compatible with automation. Using the 12 identified biomarkers, a high rate of accuracy was achieved when considering a multidimensional linear combination of features with MLR (using careful feature selection and an “all in” feature choice algorithm). While MLR with all-in feature inclusion is not the most powerful method for finding prediction models, it is most suitable for the current question, considering our sample size. Combining data of several biomarkers could be an important means of improving power of ASD diagnosis.

Some of the identified biomarkers were associated here for the first time with ASD, while others were previously described (Table 2), the latter providing external credibility for our findings and encouraging us to combine known biomarkers with the new biomarkers. Furthermore, some of the identified biomarkers are associated with various autoimmune diseases, which have been clinically associated with ASD in children, hence may support the claimed link between a subgroup of ASD cases and autoimmunity (4). A recent publication reported on proteomic analysis of sera from 76 boys with ASD and their comparison to sera from 78 TD boys (23). The group identified nine proteins that predicted ASD with a sensitivity of 83.3% and specificity of 84.6%, with all markers associated with pathways implicated in ASD, including negative regulation of immune function. Of note, none of the biomarkers overlapped with those identified in the current study.

The identification of these objective diagnostic biomarkers may open new directions for research and development in ASD.

1. Screening. The use of a widely available, inexpensive screening tool can assist clinicians to stream children with negative results away from entering lengthy wait lists for evaluation. This will greatly reduce both delays in evaluating children identified to be at higher risk of ASD and health care costs.
2. Diagnostic markers. This array can be developed into an assay for objective biological evaluation of young children with identifiable symptoms or with a family ASD history. Early and accurate diagnosis will enable earlier intervention, e.g., immune-modulating stem cells (24), at a time when the young child’s brain is most plastic, hence the potential for improved responsiveness to therapy.
3. Etiological insights. While assessment tools such as ADOS have been the accepted standard for clinical evaluation, parallel use of these biomarkers could provide insights into ASD etiology and pathogenesis.

The data and conclusions derived from this study are limited by the multiplex ELISA methodology used as well as the limited number of analyzed sera samples. These results should be independently validated in further studies with larger numbers of sera. It should be noted that this work was only a pilot, case-control diagnostic study, with a high risk of bias. The findings should be validated in larger prospective cohorts of consecutive children suspected of ASD.

In conclusion, the biomarkers identified in this work may set the stage for an objective assay for early and accurate diagnosis of ASD. In addition, the markers may shed light on ASD etiology and pathogenesis. Future research of the identified immunologic biomarkers should focus on understanding their role in the underlying immune-pathogenesis of ASD. If the results hold true in more realistic datasets, they may lead to a paradigm shift in medical practice to incorporate biomarkers into the early diagnosis and monitoring of ASD.

## Data Availability

The raw data supporting the conclusions of this article will be made available by the authors, without undue reservation.
