# Supplementary material for: Autism spectrum disorder diagnosis using a new panel of immune- and inflammatory-related serum biomarkers: A case-control multicenter study
Source: Front Pediatr. 2023 Feb 21;11:967954. doi: 10.3389/fped.2023.967954 (PMC9989209; doi:10.3389/fped.2023.967954)
Supplement: Supplementary file 1 [file Table1.docx]

**Autism spectrum disorder diagnosis using a new panel of serum biomarkers**

## MLR equations

A.

Y=-0.13*IFNg-1.25*IL10-0.84***IL17**+0.27***TNFa**-1.70*aFGF+1.09***IL4Ra**-1.08*IL6-0.31*IL1a-0.66***RBP4**-0.33

Y=-0.22*IFNg-1.09*IL10-0.96***IL17**+0.32***TNFa**-1.76*aFGF+1.42***IL4Ra**-1.07*IL6-0.11*IL1a-0.48***TFPI**-0.34

Y=-0.23*IFNg-1.15*IL10-1.03***IL17**+0.33***TNFa**-1.59*aFGF+1.08***IL4Ra**-0.87*IL6-0.11*IL1a -0.56***TFPI** -0.34

Y=-0.08*IFNg-1.00*IL10-1.03***IL17**-0.13***TNFa**-1.54*aFGF+1.26***IL4Ra**-0.81*IL6-0.24*IL1a-0.93***Kall1^b^**-0.49

Y=+0.17*IFNg-0.69*IL10-1.16***IL17**-0.04***TNFa**-2.14*aFGF-1.07*LIGHT-0.51*IL6-0.45*IL1a -0.67*Semaphorin7A-0.68

Y=-0.16*IFNg-1.19*IL10-0.93***IL17**+0.11***TNFa**-1.44*aFGF+0.92***IL4Ra**-1.14*IL6 +1.43***Procalc^c^**-0.48***TFPI**-0.22

Y=-0.02*IFNg-1.06*IL10-0.98***IL17**+0.26***TNFa**-1.14*aFGF+0.84***IL4Ra**-1.35*IL6 +1.27***Procalc^c^**-0.66***TCPTP**-0.17

Y=-0.07*IFNg-0.92*IL10-0.89***IL17**-0.15***TNFa**-1.17*aFGF+0.80***IL4Ra**-1.39*IL6 +1.44***Procalc^c^**-0.81***TCPTP**-0.26

Y=-0.36*IFNg-1.25*IL10-1.44***IL17**+0.12***TNFa**-1.35*aFGF+0.85***IL4Ra**-1.05*Crb2^a^+1.58***Procal^c^**-0.58***Kall1^b^**-0.28

Y=-0.06*IFNg-1.63*IL10-1.09***IL17**+0.32***TNFa**-1.58*aFGF+0.79***IL4Ra**-0.52***RBP4**-0.52*IL1a -0.92***TCPTP**-0.47

B.

Y=0.001*(-0.96*IFNg-63.7*IL10-26.2***IL17**+1.77***TNFa**-0.06*aFGF +301***IL4Ra**-11.2*IL6 -7.99*IL1a-0.65***RBP4**)+6.38

Y=0.001*(-1.58*IFNg-55.2*IL10-29.9***IL17**+2.06***TNFa**-0.06*aFGF +389***IL4Ra**-11.1*IL6 -2.91*IL1a-0.24***TFPI**)+3.05

Y=0.001*(-1.67*IFNg-58.6*IL10-32.1***IL17**+2.13***TNFa**-0.06*aFGF +298***IL4Ra**-9.05*IL6 -2.81*IL1a-0.29***TFPI**)+3.37

Y=0.001*(-0.56*IFNg-50.7*IL10-32.0***IL17**-0.85***TNFa**-0.05*aFGF +348***IL4Ra**-8.38*IL6 -6.24*IL1a-4.00***Kall1^b^**)+3.53

Y=0.001*(+1.26*IFNg-35.2*IL10-36.2***IL17**-0.25***TNFa**-0.07*aFGF-15.9*LIGHT-5.33*IL6 -11.5*IL1a-0.003*Semaphorin_7A)+3.21

Y=0.001*(-1.15*IFNg-60.6*IL10-28.9***IL17**+0.69***TNFa**-0.05*aFGF +254***IL4Ra**-11.8*IL6 +2.20***Procal^c^**-0.24***TFPI**)+2.65

Y=0.001*(-0.12*IFNg-54.0*IL10-30.4***IL17**+1.66***TNFa**-0.04*aFGF +231***IL4Ra**-14.0*IL6 +1.96***Procal^c^**-0.40***TCPTP**)+1.79

Y=0.001*(-0.48*IFNg-46.9*IL10-27.7***IL17**-1.00***TNFa**-0.04*aFGF +220***IL4Ra**-14.4*IL6 +2.22***Procal^c^**-0.49***TCPTP**)+2.31

Y=0.001*(-2.62*IFNg-63.3*IL10-44.8***IL17**+0.78***TNFa**-0.05*aFGF +234***IL4Ra**-0.75*Crb2^a^+2.44***Procal^c^**-2.49***Kall1^b^**)+3.37

Y=0.001*(-0.43*IFNg-82.9*IL10-33.9***IL17**+2.05***TNFa**-0.06*aFGF +216***IL4Ra**-0.52***RBP4** -13.6*IL1a -0.56***TCPTP**)+5.85

**MLR equations.** Each equation represents the model (equation) obtained for one fold. Features included in more than one model are arbitrarily color-coded such that the same feature is always shown in the same color, but the same color is never used for different features. Each equation can be used to predict ASD or TD status. (A) The original equations, as obtained from the MLR (which was applied to Z-transformed data). (B) The equations, corrected such that they can be applied to the raw data. The coefficients are reverse-transformed so that the equations can be applied to the raw data. Coefficients are rounded in both parts of the Table. For either set of equations, the prediction of ASD or TD is obtained by calculating S=e^Y^/(1+e^Y^) where Y is the result of the equation. S≥0.5 predicts ASD, and S<0.5 predicts TD. Some gene names have been shortened (^a^CarboxypeptidaseA2; ^b^Kallikrein1; ^c^Procalcitonin).
